# Grittier and More Hopeful About the Future? A Nine-Month School-Based Longitudinal Study on Grit and Adolescent Possible Selves

**DOI:** 10.3390/ejihpe15080144

**Published:** 2025-07-22

**Authors:** Shimin Zhu, Chongzeng Bi

**Affiliations:** 1 Department of Applied Social Sciences, The Hong Kong Polytechnic University, Hong Kong, China; 2 Research Centre for Psychology and Social Development, Southwest University, Chongqing 400715, China

**Keywords:** future self, perseverance, passion, secondary school students, lockdown

## Abstract

The changes in adolescents’ visions for the future are important to adolescents’ developmental trajectories, motivation, and educational outcomes, yet understudied. This study examined the change in possible selves and its association with grit during school closure and life interruption during COVID-19. We conducted a school-based longitudinal survey among 1577 students (Mage = 13.05, *SD* = 0.86) from 12 secondary schools at the start and end of an academic year prior to and during the COVID-19 pandemic with a 9-month interval. Demographic, grit, socioeconomic status (SES), self-control, and possible selves were measured. Paired *t*-tests indicated a significant decrease in academic possible selves and strategies. Hierarchical regression analysis results show that participants with higher grit scores reported higher academic and life possible selves; in particular, the effect of grit–perseverance was stronger than grit–passion after controlling self-control. SES moderated the effect of grit–passion on academic possible selves. The current longitudinal study provides important implications for education and youth social work practice for young people growing up with the influence of the pandemic.

## 1. Introduction

Adolescence is a critical developmental stage for forming visions of the future, and the way youth and adolescents see their future influences their motivation and behaviors. Researchers have found that an adolescent’s future self is a strong contributor to motivation and self-regulation, which play a significant role in predicting school performance, dropouts, and other self-regulation behaviors (Hoyle & Sherrill, 2006). This vision can change, especially during life adjustments, such as the unprecedented changes during the COVID-19 pandemic. School closures and frequent interruptions of school life during the pandemic caused long-lasting effects on adolescents’ learning, especially for those from disadvantaged homes (Engzell et al., 2021). This experience may have influenced how adolescents see their future. However, little is known about the changes in adolescents’ academic possible selves during the COVID-19 pandemic and their associated factors after months of interrupted schooling.

Possible selves are sensitive to context (Oyserman et al., 2015) and can differ according to personality differences, such as in optimists or pessimists (Carver et al., 1994). Research has shown that individuals often respond differently to the uncertainty caused by traumatic events, such as the pandemic (Killgore et al., 2020). In adverse life experiences, grit may play an important role in an individual’s psychology (Cheung et al., 2021). Grit refers to one’s persistence and passion for their goal (Duckworth et al., 2007), which may enable persistence in pursuing possible selves. However, the association between personality grit and individual differences in possible selves, especially regarding the underlying mechanism of socioeconomic situations during the pandemic, is yet to be discovered.

The present study aims to address this research gap by examining changes in adolescents’ academic possible selves and their associations with grit. We conducted a longitudinal school-based study that included a baseline survey prior to the pandemic and a follow-up survey immediately after school resumed. Our findings contribute to the understanding of adolescent development during this period and provide practical implications for education and youth services.

### 1.1. Possible Selves Among Early Adolescents

Possible selves are the future selves that a person expects, hopes, or fears to become in the near or distant future (Markus & Nurius, 1986). Possible selves are important, especially for adolescents, because having possible selves for achieving desirable outcomes is highly motivating, especially when one perceives a high likelihood of attaining the possible self and has concrete strategies (Oyserman et al., 2006). Individuals who have clear and stable future goals and believe they can achieve them are more likely to engage in self-regulatory behavior to realize those goals (Hoyle & Sherrill, 2006). Early adolescence is one of the most dynamic development stages in which possible selves and strategies shape an adolescent’s potential and the paths they take in the face of uncertainty and difficulty.

Possible selves in adolescents are the product of a complex interplay between psychological and social factors, and they are sensitive to social context (Oyserman et al., 2015). The ability to envision expansive and hopeful future selves may be attributed in part to the experiences of young people’s present lives (Prince, 2014), and the literature shows that many factors contribute to individual differences in possible selves, such as gender, age, socioeconomic status (SES), and social context (e.g., Bi & Oyserman, 2015; Knox et al., 2000; Prince, 2014; Zhu et al., 2014). However, few longitudinal investigations have examined the development of adolescents’ possible selves, particularly regarding whether it changes or remains stable over time in natural settings. In addition, the associated factors affecting this process remain largely unexplored and require both theoretical and empirical attention (Horowitz et al., 2020; J. Lee, 2021).

During the COVID-19 pandemic, life changes and school closures presented students with unique challenges, which led to increased uncertainty and difficulties in their studies and lives. Approximately 144 countries closed their schools to contain the transmission of the virus, and more than 1.2 billion students around the world were “out of the classroom” because of social distancing measures (United Nations Educational Scientific and Cultural Organization, 2021). This sudden transformation might have induced changes in students’ possible selves, influencing their confidence towards their academic potential, and the increase in online teaching may have also added extra challenges. Considering the potentially large impact of COVID-19 on adolescents’ development, it is worthwhile to examine possible selves among young people during the pandemic to advance the understanding of this topic during major life changes. Given that little is known about this subject, this study can provide parents and educators with information about the impact of COVID-19 on adolescents and help teachers, school counselors, and social workers to plan interventions.

### 1.2. Grit and Possible Selves

Grit is a personality trait that has been characterized as the capacity to sustain effort and interest in projects that take months or even longer to complete (Duckworth & Quinn, 2009). An individual’s personality trait of grit plays a role in predicting persistent goal pursuit and achievement, especially education-related goals (Allen et al., 2021; Chen et al., 2020; Fernandez-Martin et al., 2020). Grittier students are more likely to be engaged in academic studies (Datu, 2021) and, in turn, have higher academic achievements (S. Lee & Sohn, 2017). Grit may also play a crucial role in pursuing goal-oriented behaviors under uncertainty and difficulty. Duckworth and Gross (2014) found that people with a high level of grit did not become tired of achieving goals and were more confident and persistent towards their future goals. Although some research has suggested that grit is associated with possible selves on a conceptual level, the longitudinal association between grit and possible selves remains unclear.

Grit may not only be associated with a person’s vision of their future and their confidence to attain it but also be linked to their strategies for action and implementation. For example, researchers found that persistent academic possible selves promoted future achievement, but only when it was mediated by current self-regulation (J. Lee, 2021). Adolescents had been facing unprecedented challenges, such as life-threatening diseases, and they experienced difficulties in accessing resources in online study and distractions when studying at home. It can be vital to have strategies and grit to keep using valid approaches to attain possible selves. Students with greater grit may be more likely than those with lower levels of grit to adhere to strategies to realize a goal, especially when experiencing a lack of tangible support from their school.

Researchers in the field of possible selves generally agree that both possible selves and the related strategies for action are important for academic outcomes, which might also be context sensitive, according to Horowitz and colleagues (2020). Regarding COVID-19, students’ learning changed enormously (Reimers, 2022), and individual differences in personality, grit, and character in this context are interesting factors worth consideration. The processes of how strategies and self-regulation function have not been fully considered, and grit is a potentially significant factor in these processes. On the one hand, grit motivates action towards the goals set by possible selves and helps individuals follow the strategies to attain those goals. On the other hand, because of the property of persistence, it may also allow students to adhere to learning goals and strategies in the absence of a school support environment, such as the specific learning changes that emerged during the pandemic.

Grit consists of two distinct components: perseverance of effort and consistency of interest. Perseverance of effort refers to striving hard to accomplish goals despite challenges and hardships, and consistency of interest involves steady long-term interest. The effects of grit–perseverance and grit–interest are found to be different across cultures. For example, among Asian adolescents, perseverance of effort was found to significantly predict academic performance and subjective well-being, but consistency of interest did not (Datu et al., 2016). These results differ from results based on data from American samples (Duckworth et al., 2007). Thus, grit–perseverance and grit–passion may predict possible selves differently, although this relationship has not yet been examined.

Although grit and self-control are related, they are separate psychological constructs. Self-control is the capacity to regulate attention, emotion, and behavior in the presence of temptation, whereas grit is the tenacious pursuit of a dominant superordinate goal despite setbacks (Duckworth & Gross, 2014). For example, some people with high levels of self-control handle temptations well but do not consistently pursue a dominant goal. Regarding temptation during the pandemic, online learning increased adolescents’ exposure to the internet and digital devices, which may have increased temptation towards gaming and social media use (Zhu et al., 2021b). It is possible that students with higher grit and self-control adapt well in these situations and secure better academic possible selves. This study examines the association between grit and possible selves by considering the influence of self-control.

### 1.3. The Moderating Role of Socioeconomic Status

Consistent evidence has revealed a relationship between economic situation and the perceived likelihood of possible selves. Adolescents from lower SES groups reported lower expected likelihoods of obtaining their possible selves than those from higher SES groups (Zhu et al., 2014). On the other hand, researchers also found left-behind adolescents in low-SES groups were more likely to link their possible selves strategies to action to achieve future selves (Bi & Oyserman, 2015). Research has also shown that children from middle-income families are more likely to employ strategies to work on their school-focused possible identities than children from low-income families or those in low-income neighborhoods (Horowitz et al., 2020).

The COVID-19 pandemic may have exacerbated the influence of poverty on adolescent development (Patel et al., 2020). As familial financial difficulties and increased risk of unemployment often cloud young people’s views of their future, SES may have polarized the impact of COVID-19, resulting in high-SES groups experiencing more positive effects, such as more family time and support, and low-SES groups suffering more negative influences (Zhu et al., 2021a). Thus, the pandemic adversely affected most people, but the effects may be uneven. For example, online teaching might have affected students in low-SES families more than those in high-SES families, enlarging the information gaps that already existed between these two groups. High-SES families tend to seek other resources to compensate for the adverse effects of reduced teacher–student interactions and school support and the lack of operational opportunities for students to practice, especially in science-related courses (Bacher-Hicks et al., 2021; Liao et al., 2022).

However, how SES situations, especially those related to digital resources, are associated with adolescents’ possible selves is unclear, as well as the underlying interactions between SES, grit, and possible selves.

### 1.4. The Current Study

The two-wave longitudinal study was conducted prior to and during the pandemic with a nine-month interval. We examined changes in academic possible selves and how grit associates with academic and life possible selves. The current study aimed to address two research questions. First, what are the associations between grit and possible selves, and, in particular, how are the two grit components (i.e., persistence of interest and perseverance of effort) associated with possible selves? We hypothesized that higher grit would be associated with a higher likelihood of academic and life possible selves. Second, does SES moderate the association between grit and possible selves? We hypothesized that SES would moderate the association between grit and possible selves, with lower SES decreasing the association between grit and possible selves. To examine variations in the possible-self measures caused by the factor of grit, we also controlled for the factor of self-control, a close psychological construct of grit.

## 2. Materials and Methods

### 2.1. Process and Participants

This study was part of a large-scale longitudinal survey examining psychological development in secondary school students in Hong Kong. The two waves of data collection occurred at the start and end of a school year, with a nine-month interval before and during the pandemic (baseline in September 2019 and follow-up in June 2020). During the nine months, students had one semester of classroom study, from September to January, and nearly one semester of online study, from February to June 2020, because of the COVID-19 pandemic. Schools reopened briefly from 8 June until mid-July 2020, before the third wave of COVID-19 hit Hong Kong.

Before data collection, consent was obtained from the schools, parents, and students. To reduce the concern associated with being labeled, students were assured that they were volunteer participants and their teachers were not able to access their responses. Participants responded to the questionnaires in a self-administered format. To ensure the quality of the responses, two trained research assistants introduced the surveys to the students using standard instructions, answered questions, and provided guidance on how to complete the questionnaire when appropriate and without intruding on the participants. After the students finished the surveys, the research assistants packaged and sealed the questionnaires and transported them to the first author’s university. Participants can choose the English and Chinese versions of the questionnaire. Ethical approval was granted by the Human Subjects Ethics Subcommittee of the first author’s university (No. HSEARS20161222006). All participants received souvenirs (worth USD $5) after finishing the follow-up questionnaire survey.

In Wave 1 (baseline), 1721 participants (53.1% Grade 7, 46.9% Grade 8, 51.4% females) aged 10–17 years old (Mage = 12.41, *SD* = 0.82) participated. In Wave 2 (follow-up), 1577 participants, those who responded to the follow-up study, were included in the analyses (*N* = 1577). No significant differences existed between the remaining and missing participants (*N* = 144) regarding demographic and SES factors. Most participants (95%) were Chinese, 2.5% were non-Chinese, and 2.5% did not respond. Table 1 outlines the demographic characteristics of the respondents of Wave 2. In total, 345 respondents (21.9%) were from the high-SES group, 194 (12.3%) were from the low-SES group, and 930 (59%) were from the medium SES group.

### 2.2. Measures

#### 2.2.1. Measures Only in Wave 1

*Grit*. We used the eight-item Grit Scale (Duckworth & Quinn, 2009), which measures the extent to which an individual can maintain focus, interest, and perseverance when obtaining long-term goals. An example item from the passion-related subscale is “*New ideas and projects sometimes distract me from previous ones*”, and an example item from the perseverance-related subscale is “*I finish whatever I begin*”. Each item is scored on a five-point Likert scale, from 1 (*very much like me*) to 5 (*not like me at all*). The minimum score of 1 indicates that the participants have little grit, whereas the maximum score of 5 indicates they have extreme grit. The average scores of grit–perseverance and grit–passion were calculated, and Cronbach’s alpha was 0.73 and 0.75 for Waves 1 and 2, respectively.

#### 2.2.2. Measures in Both Waves

*Sociodemographic data* (including age, gender, and ethnicity). SES was measured with the Family Affluence Scale—Revised (FAS-R), which is adapted from the FAS III (Svedberg et al., 2016). The FAS is a user-friendly scale for measuring SES in child and adolescent responders. It was first developed and used by the World Health Organization’s Health Behaviour in School-aged Children project (Currie et al., 1997). The FAS-R consists of five components related to life affluence—house size (0/1), car (0/1), household appliances (0/1/2), learning devices (0/1/2), and internet accessibility (0/1/2)—and the total score ranges from 0 to 8. The composite FAS-R score categorizes responders into low (0–4), medium (5–6), and high (7–8) SES levels.

*Academic possible selves and strategies*. These factors were assessed with Kemmelmeier and Oyserman’s (2001) seven-item scale. Students rate how likely they are (0 = *not possible at all* to 9 = *very possible*) to attain three academic possible selves in the coming year (“*doing well in school*”, “*getting good grades*”, and “*understanding the material in my class*”). Cronbach’s alpha was 0.91 and 0.89 for Waves 1 and 2, respectively. Students also rate how likely they are to use four strategies to attain these academic possible selves in the coming year (“*using my time wisely*”, “*handling problems that come my way successfully*”, “*coping well with distraction*”, and “*striving persistently towards my goals*”). Cronbach’s alpha was 0.90 and 0.91 for Waves 1 and 2, respectively.

#### 2.2.3. Measures Added in Wave 2

*Life Possible Selves*. Life possible selves were measured using the adapted five-item achievement subscale of the Adolescent Life Goal Profile Scale (Gabrielsen et al., 2012). Respondents rate how likely statements about future life are to describe their future life. Sample statements include “*I will be successful and have great achievements*” and “*I will get status for something I am good at*”. Each item is scored on a ten-point scale, rating from 0 (*impossible*) to 9 (*highly possible*). The mean of the five items was taken as a measure of possible selves, with a higher score indicating a greater likelihood of fulfilling the possible life in the future. The scale demonstrated high internal consistency (*α* = 0.936).

*Self-control*. The Self-Control Scale (Tangney et al., 2004; Unger et al., 2016) was used to measure individual differences in traits of self-control. The items were scored on a six-point scale, ranging from 1 (*strongly disagree*) to 6 (*strongly agree*). The mean was used as a measure of self-control, with a higher value indicating a higher level of self-control. An example item is “*I am good at resisting temptation*”. Cronbach’s alpha was 0.783.

### 2.3. Analysis

Descriptive analysis was conducted for the demographic and study measure data. The baseline and nine-month follow-up of possible selves were compared using paired *t*-tests. Hierarchical regressions were used to test the hypotheses. In Step 1, we examined the associations of demographics and SES with possible selves. In Step 2, we examined the associations of grit with possible selves. In Step 3, we added self-control to the model and examined the association of grit with possible selves. In Step 4, we examined the moderation effect of SES and grit. In Step 5, baseline possible selves were added to the model. As baseline possible selves were hypothesized to be stable and strong predictors of possible selves, Step 5 was expected to show the coefficient changes of grit on possible selves after controlling for baseline possible selves. SPSS (Version 26) was used to analyze the data.

## 3. Results

### 3.1. Descriptive Analysis and Correlations

The means for academic-related possible selves and strategies in Wave 1 were 5.50 (*SD* = 1.91) and 5.80 (*SD* = 1.92), respectively. The possible selves measures were above the midpoint (4.5) of the scale, indicating that participants believed they were quite likely to achieve academic-related possible selves. At the end of the academic year (Wave 2), participants reported significantly lower academic-related possible selves (*Mean* = 4.87, *SD* = 1.85) and strategies (*Mean* = 5.17, *SD* = 1.88), shown by paired sample *t*-tests: *t*_APS_ = 12.96, *p* < 0.001, Cohen’s *d* = 0.33 and *t*_strategies_ = 13.27, *p* < 0.001, Cohen’s *d* = 0.34. At the end of the academic school year, respondents were less positive about the likelihood they would do well in schoolwork (see Table 2).

Correlation results showed that significant associations existed between grit and possible selves measures (see Table 3), with stronger associations between perseverance of effort and possible selves measures (*r*s = 0.34 to 0.61, *p*s < 0.001) than between persistence of interest and possible selves measures (*r*s = 0.15 to 0.25, *p*s < 0.001). As expected, both components of grit were correlated with self-control (*r*s = 0.31 and 0.39, *p*s < 0.001), supporting the rationale of controlling for the self-control measures.

### 3.2. Association Between Grit and Possible Selves and Strategies

The results of the hierarchical linear regressions revealed significant associations between grit and possible selves, with a stronger association for perseverance of effort. The results of the regressions on academic possible selves (Table 4) and on strategies (Table 5) have similar patterns. In Step 2, grit–interest and grit–perseverance were significantly associated with possible selves measures, but the coefficients of grit–interest (betas = 0.08 to 0.13, *p*s < 0.05) were much lower than those of grit–perseverance (betas = 0.32 to 0.42, *p*s < 0.001). After controlling for self-control in Step 3, the coefficients of both grit components dropped, among which grit–perseverance was still significant (betas = 0.22 to 0.28, *p*s < 0.001), but grit–interest was not (betas = 0.00 to 0.03, *p*s > 0.05).

### 3.3. Moderation Effects of SES

**Figure 1:**
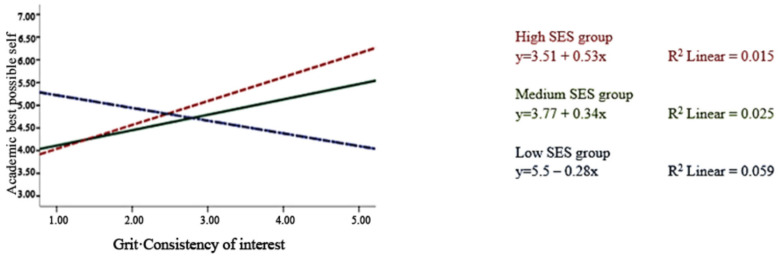
Moderation effect of the socioeconomic status factor on the association between grit–interest and academic possible selves.

**Figure 2:**
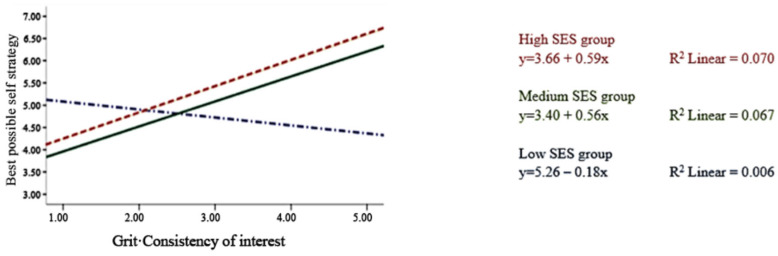
Moderation effect of the socioeconomic status factor on the association between grit–interest and academic possible selves strategies.

Moderation effects of SES were found on academic possible selves and strategies, but not on life possible selves. The moderation effects of SES on grit–interest were stronger than those of grit–perseverance on academic possible selves and strategy: beta of grit–interest on academic possible selves = 0.08, *p* < 0.05; beta of grit–perseverance on academic possible selves = −0.01, *p* > 0.05; beta of grit–interest on strategy = 0.07, *p* < 0.01; and beta of grit–perseverance on strategy = −0.05, *p* < 0.05). Figure 1 and Figure 2 show the interaction effects. Among the three groups of SES, the association between grit–interest and possible selves differed by SES.

In Step 5, after controlling for baseline possible selves measures, the association of grit–perseverance and strategy was still significant (beta = 0.10, *p* < 0.001), and the moderations of SES on the association between grit–interest and academic possible selves (beta = 0.08, *p* < 0.001) and strategy (beta = 0.06, *p* < 0.01) were still significant. Respondents with higher SES had higher possible selves and strategy while having higher grit of consistency of interest.

A similar effect was found in the association with grit and life possible selves (Table 6). Grit of perseverance was positively associated with life possible selves after controlling for self-control. No interaction effect was found between SES and grit on life possible selves.

## 4. Discussion

This study on early adolescents from before to during the COVID-19 pandemic examined the change in academic possible selves and its association with grit. The results revealed a significant decrease in the perceived likelihood of academic possible selves and strategies at the end of the academic year, indicating that grit–perseverance is a much stronger contributory factor to both academic and life possible selves measures than grit–passion is. We also found interaction effects of SES on the association between grit–passion and possible selves measures, but not on the association between grit–perseverance and possible selves.

### 4.1. Association Between Grit and Possible Selves

This decrease in academic possible selves during the pandemic deserves attention. Despite a previous finding that the perceived likelihood of possible selves increased across one semester (Dunkel & Anthis, 2001), the decrease in possible selves and strategies we found across one academic year was consistent with Horowitz and colleagues’ findings (2020). Horowitz et al. (2020) found that school-focused possible identities among 8th graders decline over the school year, and the decline is associated with declining academic trajectories. In addition, the decrease in possible selves may be beyond a mere academic-year effect, possibly reflecting a profound impact of the COVID-19 pandemic on adolescent development. In fact, the pandemic likely caused general long-term impacts on adolescent development (United Nations Educational Scientific and Cultural Organization, 2021). The adaptation to online learning, greater demand for self-regulation because of school closures, more temptations towards gaming, greater uncertainty about the future, and less tangible school support all posed students with unprecedented challenges. For instance, studies have indicated that the perceived support and communication from teachers played significant roles in adolescents’ expressing concrete action plans to achieve their expected possible selves (Mireles-Rios & Roshandel, 2020). The change in teaching mode during the pandemic led to a lack of interactions with and support from teachers, which might have contributed significantly to the decreases in adolescents’ possible selves. Thus, more helpful and supportive programs should be needed to help students stay hopeful about attaining academic goals and feel competent to overcome difficulties in schoolwork after challenging situations subside. Otherwise, in the long run, students may risk falling behind and losing motivation, leading to school dropout or passively coping with academic and life difficulties.

We also found a longitudinal association between grit and academic possible selves. Personal attributes play a role in the mechanisms of possible selves formation and change (e.g., Carver et al., 1994), and this is the first study to provide empirical findings on how grit is associated with possible selves after one year. In this study, the associations of grit–perseverance with both academic and life possible selves were much higher than those of grit–passion. This may be because grit–perseverance is more highly emphasized than consistency of interest in Chinese culture (Datu et al., 2016). Consistent with previous studies, this finding indicates that grit–perseverance is closely related to academic goals (Tang et al., 2021; Usher et al., 2019). Adolescents with higher perseverance of effort are more confident that they can achieve better life possible selves, academic possible selves, and strategy. Given that attaining possible selves requires long-term effort and persistence, grit–perseverance plays an important role.

### 4.2. Moderation Effects of SES

Regarding SES, we found that the associations between grit–passion and academic possible selves were moderated by SES. In particular, participants in the low-SES group reported lower academic and life possible selves, along with an increase in grit–passion. Previous research found that low-SES students were less likely to believe they could attain their possible selves (Zhu et al., 2014). The current research may further explain the possible reason. Persisting in interests, such as sports and music, may involve spending more time and resources on pursuing those interests rather than on academic-related activities. With limited resources, students in lower SES groups may lower their perceived likelihood of their academic possible selves. Moreover, the pandemic likely widened achievement gaps along these dimensions, given schools’ and parents’ differing engagement levels with online resources to compensate for lost school-based learning time (Bacher-Hicks et al., 2021). Students in high-need districts were significantly less likely to complete their work (Catalano et al., 2021). As low-SES students rely more than others on school education, they experience a more profound impact than higher-SES groups. It is imperative to realize that grit can affect possible selves independent of self-control, as shown by our study, indicating that tailored interventions can be made to counteract the negative effects of the pandemic on adolescents, especially for those with low-SES situations (Bacher-Hicks et al., 2021; Catalano et al., 2021; van de Werfhorst et al., 2020).

This investigation is one of the few longitudinal school-based studies that has tracked changes in possible selves and the longitudinal association between grit and possible selves in a large early adolescent sample. The prospective cohort nature of this study enabled us to examine factors that potentially relate to changes in possible selves and the longitudinal association between grit and possible selves before and during the pandemic. This study has significant implications. Adolescents’ possible selves play a significant role in adolescent development. The unprecedented interruptions caused by the COVID-19 pandemic may have influenced adolescents’ envisioning about the future, especially for adolescents from low-SES backgrounds (Bacher-Hicks et al., 2021). The significant decrease we found in academic possible selves and strategies highlights the need for remedial actions to reduce the negative impact on adolescents’ hope and strategies for achieving academic goals and to boost their confidence in pursuing goals. We also found that grit is a salient personality factor that contributes to individual differences in adolescents’ possible selves. Therefore, interventions to foster grit, in particular perseverance, would help adolescents to maintain efforts to achieve possible selves during challenging and difficult times. In addition, specific vulnerable groups of young people who are already experiencing inequalities (e.g., poor family support and poverty) and those who had poor physical and mental health before the outbreak are likely to become more vulnerable (Holmes et al., 2020). Students from low-SES families should be prioritized for receiving support when schools are closed and reopened. To allow these young people to contribute to society in the future, governments, policymakers, and leaders in education, health, and social services need to anticipate and prepare for similar emerging situations and intervene at an early stage to prevent the loss of skills and resources.

### 4.3. Strengths and Limitations

The current study has both strengths and limitations. The two-wave classroom survey study among a large sample of secondary school students allows a rigorous examination of the longitudinal association between grit and possible selves and strategies. It clarified the intricate relation between the two grit components and the near and distant possible selves. However, it also has limitations. First, the self-reported measures may be subject to social desirability bias. Second, although the hierarchical regression analyses controlled baseline data, this method does not permit causal inferences or the determination of bi-directional relationships between grit and possible selves. To address these limitations, future studies may employ more advanced longitudinal analytic methods, such as cross-lagged panel models or random intercept cross-lagged models using three-wave longitudinal data to examine the bidirectional relation between possible selves and grit.

## 5. Conclusions

The changes in possible selves of adolescents in life adjustment during the COVID-19 pandemic shed light on the post-pandemic developmental trajectory. This two-wave longitudinal study on secondary school students found that possible selves decreased during the pandemic, and grit was associated with life and academic possible selves. The grittier in perseverance, the more positive about the academic future. The effect of grit–perseverance was stronger than that of grit–passion. Moreover, SES moderated the effect of grit–passion on academic possible selves. Students from lower SES may have high grit in passion but low belief in attaining their future selves. Remedial actions are needed to reduce the negative impact of the pandemic on adolescents’ hope and strategies for achieving academic goals and to boost their confidence in pursuing goals, especially for those who are disadvantaged. It is essential for designing interventions for promoting positive future orientation among adolescents in the post-pandemic era.

## Figures and Tables

**Table 1:** Social demographic information (*N* = 1577).

		*N*	%
Gender	Male	734	46.5
	Female	843	53.5
Grade	Grade 7	838	53.1
	Grade 8	739	46.9
Ethnic	Chinese	1499	95.05
	Non-Chinese	39	2.47
	Did not respond	39	2.47
SES	Low	194	12.3
	Medium	930	59.0
	High	345	21.9
	Did not identify	108	6.8
		***Mean***	***SD***
Age	Range of 10–16 years	13.05	0.86

**Table 2:** Paired *t*-test of possible selves measures reported in Wave 1 and Wave 2.

	Wave 1		Wave 2		*t*	*Cohen’ d*
	*Mean*	*SD*	*Mean*	*SD*		
1-year possible selves						
Academic possible self	5.50	1.91	4.87	1.85	12.96 ***	0.33
Possible self strategy	5.80	1.92	5.17	1.88	13.27 ***	0.34
10-year possible selves						
Best possible life			4.24	2.02		

Note. *** *p* < 0.001.

**Table 3:** The descriptive statistics and Pearson correlation.

	Range	*Mean*	*SD*	Pearson Correlation							
				1	2	3	4	5	6	7	8
1. Grit—Consistency of interest	1–5	3.19	0.85	-							
2. Grit—Perseverance of effort	1–5	3.23	0.78	0.19 ***	-						
3. Baseline APS	0–9	5.50	1.91	0.17 ***	0.54 ***	-					
4. Baseline APS strategy	0–9	5.80	1.92	0.25 ***	0.61 ***	0.77 ***	-				
5. Self-control	1–6	3.66	0.78	0.31 ***	0.39 ***	0.28 ***	0.34 ***	-			
6. APS	0–9	4.87	1.85	0.15 ***	0.34 ***	0.50 ***	0.43 ***	0.38 ***	-		
7. APS strategy	0–9	5.17	1.88	0.21 ***	0.44 ***	0.50 ***	0.52 ***	0.50 ***	0.78 ***	-	
8. Life possible self	0–9	4.24	2.02	0.12 ***	0.36 ***	0.47 ***	0.45 ***	0.30 ***	0.64 ***	0.62 ***	-

Note. APS = academic possible selves; *** *p* < 0.001.

**Table 4:** Hierarchical linear regression on the association of grit and Wave 2 academic possible self.

	Step 1		Step 2		Step 3		Step 4		Step 5	
	β	Δ R^2^	β	Δ R^2^	β	Δ R^2^	β	Δ R^2^	β	Δ R^2^
Gender	−0.00	0.02 ***	0.00	0.12 ***	0.03	0.07 ***	0.03	0.01 **	0.03	0.12 ***
Age	0.06 *		0.06 *		0.06 **		0.06 *		0.03	
SES	0.11 ***		0.07 **		0.06 **		0.06 *		0.04	
Grit—Consistency of interest			0.08 **		0.00		0.00		−0.02	
Grit—Perseverance of effort			0.32 ***		0.22 ***		0.22 ***		0.01	
Self-control					0.30 ***		0.30 ***		0.27 ***	
Grit—Consistency of interest * SES							0.08 **		0.08 ***	
Grit—Perseverance of effort * SES							−0.01		−0.00	
Baseline academic possible self									0.42 ***	

Note. * *p* < 0.05, ** *p* < 0.01, *** *p* < 0.001.

**Table 5:** Hierarchical linear regression on the association of grit and Wave 2 possible self strategy.

	Step 1		Step 2		Step 3		Step 4		Step 5	
	β	Δ R^2^	β	Δ R^2^	β	Δ R^2^	β	Δ R^2^	β	Δ R^2^
Gender	0.02	0.02 ***	0.03	0.21 ***	0.06 **	0.12 ***	0.06 **	0.01 ***	0.06 **	0.07 ***
Age	0.03		0.03		0.03		0.03		0.03	
SES	0.14 ***		0.09 ***		0.08 ***		0.07 ***		0.07 **	
Grit—Consistency of interest			0.13 ***		0.03		0.03		−0.01	
Grit—Perseverance of effort			0.42 ***		0.28 ***		0.28 ***		0.10 ***	
Self-control					0.39 ***		0.39 ***		0.36 ***	
Grit—Consistency of interest * SES							0.07 **		0.06 **	
Grit—Perseverance of effort * SES							−0.05 *		−0.03	
Baseline possible self strategy									0.33 ***	

Note. * *p* < 0.05, ** *p* < 0.01, *** *p* < 0.001.

**Table 6:** Hierarchical linear regression on the association of grit and life possible selves.

	Step 1		Step 2		Step 3		Step 4	
	β	Δ R^2^	β	Δ R^2^	β	Δ R^2^	β	Δ R^2^
Gender	0.00	0.02 ***	0.01	0.12 ***	0.02	0.03 ***	0.02	0.00
Age	0.08 **		0.09 ***		0.09 ***		0.08 ***	
SES	0.12 ***		0.08 ***		0.08 **		0.07 **	
Grit—Consistency of interest			0.05 *		0.01		0.00	
Grit—Perseverance of effort			0.34 ***		0.27 ***		0.27 ***	
Self-control					0.20 ***		0.19 ***	
Grit—Consistency of interest * SES							0.05	
Grit—Perseverance of effort * SES							0.02	

Note. * *p* < 0.05, ** *p* < 0.01, *** *p* < 0.001.

## Data Availability

The raw data supporting the conclusions of this article will be made available by the authors on request.

## Abbreviations

The following abbreviations are used in this manuscript:

SES	socioeconomic status
FAS-R	Family Affluence Scale—Revised
APS	academic possible selves

## References

[B1-ejihpe-15-00144] Allen R. E., Kannangara C., Carson J. (2021). True grit: How important is the concept of grit for education? A narrative literature review. International Journal of Educational Psychology: IJEP.

[B2-ejihpe-15-00144] Bacher-Hicks A., Goodman J., Mulhern C. (2021). Inequality in household adaptation to schooling shocks: Covid-induced online learning engagement in real time. Journal of Public Economics.

[B3-ejihpe-15-00144] Bi C., Oyserman D. (2015). Left behind or moving forward? Effects of possible selves and strategies to attain them among rural Chinese children. Journal of Adolescence.

[B4-ejihpe-15-00144] Carver C. S., Reynolds S. L., Scheier M. F. (1994). The possible selves of optimists and pessimists. Journal of Research in Personality.

[B5-ejihpe-15-00144] Catalano A. J., Torff B., Anderson K. S. (2021). Transitioning to online learning during the COVID-19 pandemic: Differences in access and participation among students in disadvantaged school districts. The International Journal of Information and Learning Technology.

[B6-ejihpe-15-00144] Chen P., Powers J. T., Katragadda K. R., Cohen G. L., Dweck C. S. (2020). A strategic mindset: An orientation toward strategic behavior during goal pursuit. Proceedings of the National Academy of Sciences.

[B7-ejihpe-15-00144] Cheung S. N., Huang C. C., Zhang C. C. (2021). Passion and persistence: Investigating the relationship between adverse childhood experiences and grit in college students in China. Frontiers in Psychology.

[B8-ejihpe-15-00144] Currie C. E., Elton R. A., Todd J., Platt S. (1997). Indicators of socioeconomic status for adolescents. Health Education Research.

[B9-ejihpe-15-00144] Datu J. A. D. (2021). Beyond passion and perseverance: Review and future research initiatives on the science of grit. Frontiers in Psychology.

[B10-ejihpe-15-00144] Datu J. A. D., Valdez J. P. M., King R. B. (2016). Perseverance counts but consistency does not! Validating the short grit scale in a collectivist setting. Current Psychology.

[B11-ejihpe-15-00144] Duckworth A. L., Gross J. J. (2014). Self-control and grit: Related but separable determinants of success. Current Directions in Psychological Science.

[B12-ejihpe-15-00144] Duckworth A. L., Peterson C., Matthews M. D., Kelly D. R. (2007). Grit: Perseverance and passion for long-term goals. Journal of Personality and Social Psychology.

[B13-ejihpe-15-00144] Duckworth A. L., Quinn P. D. (2009). Development and validation of the Short Grit Scale (Grit-S). Journal of Personality Assessment.

[B14-ejihpe-15-00144] Dunkel C. S., Anthis K. S. (2001). The role of possible selves in identity formation: A short-term longitudinal study. Journal of Adolescence.

[B15-ejihpe-15-00144] Engzell P., Frey A., Verhagen M. D. (2021). Learning loss due to school closures during the COVID-19 pandemic. Proceedings of the National Academy of Sciences.

[B16-ejihpe-15-00144] Fernandez-Martin F. D., Arco-Tirado J. L., Hervas-Torres M. (2020). Grit as a predictor and outcome of educational, professional, and personal success: A systematic review. Psicologia Educativa.

[B17-ejihpe-15-00144] Gabrielsen L. E., Ulleberg P., Watten R. G. (2012). The adolescent life goal profile scale: Development of a new scale for measurements of life goals among young people. Journal of Happiness Studies.

[B18-ejihpe-15-00144] Holmes E. A., O’Connor R. C., Perry V. H., Tracey I., Wessely S., Arseneault L., Ballard C., Christensen H., Cohen Silver R., Everall I., Ford T., John A., Kabir T., King K., Madan I., Michie S., Przybylski A. K., Shafran R., Sweeney A., Bullmore E. (2020). Multidisciplinary research priorities for the COVID-19 pandemic: A call for action for mental health science. The Lancet Psychiatry.

[B19-ejihpe-15-00144] Horowitz E., Oyserman D., Dehghani M., Sorensen N. (2020). Do you need a roadmap or can someone give you directions: When school-focused possible identities change so do academic trajectories. Journal of Adolescence.

[B20-ejihpe-15-00144] Hoyle R. H., Sherrill M. R. (2006). Future orientation in the self-system: Possible selves, self-regulation, and behavior. Journal of Personality.

[B21-ejihpe-15-00144] Kemmelmeier M., Oyserman D. (2001). Gendered influence of downward social comparisons on current and possible selves. Journal of Social Issues.

[B22-ejihpe-15-00144] Killgore W. D., Taylor E. C., Cloonan S. A., Dailey N. S. (2020). Psychological resilience during the COVID-19 lockdown. Psychiatry Research.

[B23-ejihpe-15-00144] Knox M. S., Funk J., Elliott R., Bush E. G. (2000). Gender differences in adolescents’ possible selves. Youth and Society.

[B24-ejihpe-15-00144] Lee J. (2021). Unveiling the relationships among adolescents’ persistent academic possible selves, academic self-concept, self-regulation, and achievement: A longitudinal and moderated mediation study. Self and Identity.

[B25-ejihpe-15-00144] Lee S., Sohn Y. W. (2017). Effects of grit on academic achievement and career-related attitudes of college students in Korea. Social Behavior and Personality.

[B26-ejihpe-15-00144] Liao H., Ma S., Xue H. (2022). Does school shutdown increase inequality in academic performance? Evidence from COVID-19 pandemic in China. China Economic Review.

[B27-ejihpe-15-00144] Markus H., Nurius P. (1986). Possible selves. American Psychologist.

[B28-ejihpe-15-00144] Mireles-Rios R., Roshandel S. (2020). Perceived teacher support and communication in strategizing possible selves. Frontiers in Education.

[B29-ejihpe-15-00144] Oyserman D., Bybee D., Terry K. (2006). Possible selves and academic outcomes: How and when possible selves impel action. Journal of Personality and Social Psychology.

[B30-ejihpe-15-00144] Oyserman D., Destin M., Novin S. (2015). The context-sensitive future self: Possible selves motivate in context, not otherwise. Self and Identity.

[B31-ejihpe-15-00144] Patel J., Nielsen F., Badiani A., Assi S., Unadkat V., Patel B., Ravindrane R., Wardle H. (2020). Poverty, inequality and COVID-19: The forgotten vulnerable. Public Health.

[B32-ejihpe-15-00144] Prince D. (2014). What about place? Considering the role of physical environment on youth imagining of future possible selves. Journal of Youth Studies.

[B33-ejihpe-15-00144] Reimers F. M. (2022). Primary and secondary education during COVID-19: Disruptions to educational opportunity during a pandemic.

[B34-ejihpe-15-00144] Svedberg P., Nygren J. M., Staland-Nyman C., Nyholm M. (2016). The validity of socioeconomic status measures among adolescents based on self-reported information about parents occupations, FAS and perceived SES; implication for health related quality of life studies. BMC Medical Research Methodology.

[B35-ejihpe-15-00144] Tang X., Wang M.-T., Parada F., Salmela-Aro K. (2021). Putting the goal back into grit: Academic goal commitment, grit, and academic achievement. Journal of Youth and Adolescence.

[B36-ejihpe-15-00144] Tangney J. P., Baumeister R. F., Boone A. L. (2004). High self-control predicts good adjustment, less pathology, better grades, and interpersonal success. Journal of Personality.

[B37-ejihpe-15-00144] Unger A., Bi C., Xiao Y. Y., Ybarra O. (2016). The revising of the Tangney self-control scale for Chinese students. PsyCh Journal.

[B38-ejihpe-15-00144] United Nations Educational Scientific and Cultural Organization (2021). Global monitoring of school closures caused by COVID-19.

[B39-ejihpe-15-00144] Usher E. L., Li C. H. R., Butz A. R., Rojas J. P. (2019). Perseverant grit and self-efficacy: Are both essential for children’ academic success?. Journal of Educational Psychology.

[B40-ejihpe-15-00144] van de Werfhorst H., Kessenich E., Geven S. (2020). The digital divide in online education. Inequality in digital preparedness of students and schools before the start of the COVID-19 pandemic. SocArXiv Papers.

[B41-ejihpe-15-00144] Zhu S., Tse S., Cheung S. H., Oyserman D. (2014). Will I get there? Effects of parental support on children’s possible selves. British Journal of Educational Psychology: Special Issue Parents’ Role in Children’s School Lives: Student Motivation and Socio-Emotional Functioning.

[B42-ejihpe-15-00144] Zhu S., Zhuang Y., Ip P. (2021a). Impacts on children and adolescents’ lifestyle, social support and their association with negative impacts of the COVID-19 pandemic. International Journal of Environmental Research and Public Health.

[B43-ejihpe-15-00144] Zhu S., Zhuang Y., Lee P., Li J. C.-M., Wong P. W. (2021b). Leisure and problem gaming behaviors among children and adolescents during school closures caused by COVID-19 in Hong Kong: Quantitative cross-sectional survey study. JMIR Serious Games.

